# Social and Emotional Loneliness in Older Community Dwelling-Individuals: The Role of Socio-Demographics

**DOI:** 10.3390/ijerph192416622

**Published:** 2022-12-10

**Authors:** Vanessa Ibáñez-del Valle, Silvia Corchón, Georgiana Zaharia, Omar Cauli

**Affiliations:** 1 Frailty Research Organized Group (FROG), University of Valencia, 46010 Valencia, Spain; 2 Department of Nursing, University of Valencia, 46010 Valencia, Spain

**Keywords:** loneliness, emotional loneliness, social loneliness, elderly, community dwelling-individuals

## Abstract

Background: Social determinants have a major influence on individuals’ health, and among them, loneliness has an important impact on the health of the elderly. Objectives: The aims were to determine loneliness and its social and emotional components in a sample of elderly people and to assess its prevalence and associations with sociodemographic variables. Methods: Analytical, cross-sectional, observational research was carried out based on a population over 60 years of age in Valencia (Spain). Loneliness was assessed with the De Jong-Gierveld Loneliness Scale. Results: Five-hundred and thirty community-dwelling individuals participated. The mean age of the sample was 72.7 years (84.2% women); 36.2% suffered from moderate loneliness and 6.6% suffered from extreme loneliness. The sociodemographic variables most significantly related to loneliness were being single, separated, or divorced (*p* < 0.01). Among widowers, loneliness was inversely associated with years of widowhood (*p* < 0.01). Having sons/daughters was a significant protective factor (*p* < 0.05), while having grandchildren or siblings did not have a significant influence. The ability to walk and smartphone and video call use were not associated with loneliness. Conclusions: There is a high prevalence of unwanted loneliness in community-dwelling individuals, and some social factors play an important role. Interventions against loneliness among older people are a priority for welfare and public health.

## 1. Introduction

Recent research indicates that all countries in the world are likely to experience increases in their population due to the increased life expectancy [1]. By 2040, Spain will be the country that ranks first, with an average life expectancy of 85.8 years [1]. Loneliness is a risk factor for morbidity and mortality, and older people are more vulnerable to feeling alone due to age-associated changes and losses that they might experience [2]. Therefore, the aging of the population is one of the most significant social changes of the twenty-first century, which requires additional resources in social and healthcare systems to guarantee and improve this population’s well-being and quality of life.

Loneliness is an unpleasant experience or feeling associated with a lack of close relationships [3]. According to Valtorta and Hanratty (2016) [4], it is a subjective negative feeling associated with the lack of a broader social network (social loneliness) or the absence of desired company (emotional loneliness). In line with Weiss (1973) [5], social loneliness refers to a deficit in a person’s social relationships, social network, and social support, while emotional loneliness is a lack of closeness or intimacy with another.

Loneliness and social isolation are considered public health concerns [6]. Health problems and certain sociodemographic conditions associated with aging, such as the death of spouses and partners, an increased likelihood of living alone, and having fewer trusting relationships, make older people particularly vulnerable to loneliness and social isolation [7]. Various studies have determined that loneliness and social isolation are significant problems for older adults that have adverse consequences for their mental and physical health [8,9,10]. Studies, such as the one by Martín-María et al. (2020) [11], show that people with chronic loneliness have poorer health. Other research, such as that of Tomás et al. (2019) [12], confirms a negative association between loneliness and life satisfaction and a positive association between loneliness and depression [6], increased perceived stress [13], and sleep disturbances [14], among other problems.

Environmental factors, such as social isolation, having a small social network, or having little participation in activities with others, are associated with cognitive decline and Alzheimer’s disease. In contrast, having an extensive social network and a large social support network are associated with less cognitive decline in old age [11]. Some authors have focused on studying emotional predictors and have shown an association between sensitivity to psychological distress and loneliness and an increased risk of dementia with old age [15,16].

Cacioppo et al. [17] documented the association between loneliness and depression, demonstrating a strong association among older adults. They also observed that loneliness and depressive symptomatology might synergistically decrease well-being in older adults. However, authors, such as Domènech-Abella et al. [18], point out that whether it is loneliness that causes depression, whether it is depression that increases feelings of loneliness, or both, has not been clearly established. Erzen and Çikrikci [19] conducted a study to determine the effect of loneliness on depression. The results of the meta-analysis that they conducted show that loneliness has a significantly impact, at moderate levels, on depression. The sample group analysis showed that lone caregivers have greater depressive tendencies, and similar results were identified for patients, students, and the elderly.

Loneliness is a multidimensional concept [20] that affects people at different times in their life cycle. It is dynamic, and as a feeling, it can affect even those who are not physically alone. There are currently two conceptual approaches to loneliness. One of them considers loneliness a unitary state that varies only in intensity as a consequence of the deficits within different relationships. The other concept, first proposed by Weiss in 1973 [5], distinguishes between two types of loneliness: social loneliness and emotional loneliness. In his definition, Weiss calls emotional loneliness the absence of intimate bonds with other people and relates social loneliness to the absence of a social support network or the lack of social integration. Weiss differentiates conceptually, for the first time, between social loneliness and emotional loneliness. He describes social loneliness as the lack or insufficiency of relationships or a sense of community and emotional loneliness as the absence of intimate personal relationships or attachment.

There have been few studies to date that distinguish between these two dimensions of loneliness [21]. Still, in a recent study [21] conducted on older adult and community-dwelling participants, older age was associated with both greater emotional loneliness and with greater social loneliness.

In addition to age, other psychosocial risk factors lead to unwanted loneliness. Knowing them is the first step to detecting who is potentially more vulnerable to unwanted loneliness and preventing it. Some of them, such as gender, marital status, or loss of attachment relationships, have been studied. In general, it is women who report feeling lonelier. The study by Fierloos et al. (2021) [22] revealed that women who live with a partner are more prone to emotional loneliness than men who live with a partner. Age, living without a partner, and having a low level of education were found to be associated with greater social loneliness. Similarly, according to Domènech-Abella et al. (2017) [18], feelings of loneliness are more prevalent in women. Other studies, such as that of Pagan et al. (2020) [23], revealed an increase in loneliness regardless of gender. According to a recent study, loneliness increases steadily from the age of 74 for both men and women and reaches a peak at around the age of 95 years [23].

Regarding other types of socio-family variables, there is no strong evidence for a relationship between unwanted loneliness and other factors that could be protective and that are currently present in society due to new role changes in households. Living with children due to unemployment or divorce or caring for grandchildren could be some of these protective factors. There are hardly any studies looking at the use of new technologies as protective tools against loneliness [21,24,25]. There is no relevant research on community-dwelling older adults that shows the relationship between feelings of loneliness and the use of telephones or smartphones to make video calls or engage in social contact through virtual social networks.

The objectives of this study were to assess the prevalence of unwanted loneliness and the subdimensions of social and emotional loneliness in community-dwelling older individuals, as well as to identify the family and social determinants that play a significant role in loneliness.

## 2. Materials and Methods

### 2.1. Design of the Study and Population

The research project was based on an analytical, cross-sectional, and observational primary study. The study population was made up of individuals over 60 years of age in Valencia and in the municipality of Moncada, for reasons of accessibility of the samples. The principal researcher of the present study was the director of the Chair of Healthy, Active and Participative Ageing at the University of Valencia, in collaboration with the Active Ageing Department of Valencia City Council (Spain), through the Municipal Centers of Activities for Older Adults in Valencia (CMAPM).

### 2.2. Study Population

The study population or target population was made up of people over 60 years of age integrated in the community in the year 2021–2022 attending various centers for activities and leisure time for older adults in Valencia (Spain). These centers are run by Valencia City Council’s Active Ageing Department. The subjects were obtained by means of a non-probabilistic convenience sampling method, in which participants were selected according to their accessibility. Taking into account the characteristics of the study population, participants were involved in activities for older adults in the Centros de Mayores of the City Hall of Valencia, with 85.5% (data recorded in 2021) of them women. The sampling in the study was formed using a similar percentage of women e.g., 84.2%, which is very similar to the study population. The sample size was calculated using the population estimation, considering the total number of older adults doing the activities (attending workshops and activities in municipal centers for older people) in Valencia City Council’s centers (*N* = 901 June 2021–June 2022). According to the calculations, a minimum sample size of 312 randomly selected subjects was determined to be sufficient for estimations with 95% confidence and a precision +/− 0.99 units, considering a standard deviation of 11 units (since the score of the loneliness scale range is between 0 and 11 points). The final sampling was formed using 530 individuals, which exceeded 218 cases, allowing us to make an acceptable estimate of the prevalence of loneliness. The subjects comprised individuals aged 60 years and over, because they have access to the activities available at these municipal centers from this age onwards. The inclusion criteria were therefore: age 60 years old and over, being a community-dwelling individual, being able to perform basic activities of daily life, with adequate cognitive function to understand and answer the questionnaire, and being able to understand Spanish.

### 2.3. Information Collection Procedure

For the purposes of data collection, an assessment instrument composed of the De Jong-Gierveld Loneliness Scale [26] was administered in order to evaluate the degree of emotional and social loneliness. This scale has been validated in Spanish [27]. In addition, socio-demographic data were collected, as well as data on the respondents’ mobility, such as a cane or walking stick, a walker or a wheelchair, housing (on which floor do you live? do you have an elevator?), and their use of smartphones and apps. The data were collected by administering an anonymous questionnaire on socio-demographic factors and the Loneliness scale to older adults participating in workshops in the *Centros de Actividades para Personas Mayores* (Senior Citizens Activity Centers) run by the Active Ageing department of Valencia City Council. At the end of the questionnaire, we asked if the participants wished to be contacted by phone in order to obtain help to overcome their loneliness.

#### Evaluation of Emotional and Social Loneliness

The De Jong-Gierveld Loneliness Scale has probably been the most widely used instrument for quantifying the construct of loneliness in a European context, because it is not culturally biased [28]. Its main advantages and the reason why it has been chosen for this study are its simplicity and brevity, as well as its good psychometric properties with older adults [29]. In addition, the final translation into Spanish has been approved by expert psychologists [28]. The De Jong-Gierveld Loneliness Scale assesses loneliness in a two-dimensional way, considering both the social dimension (lack of close emotional ties) and the emotional dimension (subjective lack of a belonging network) [29]. This scale is a questionnaire with 11 items. Six of these items (numbers 2, 3, 5, 6, 9, and 10) ask about the emotions caused by the absence of close social ties using a negative formulation, thereby assessing social loneliness. The remaining five items (1, 4, 7, 8, and 11) assess emotional loneliness. They are formulated in positive terms and ask about the respondent’s close social ties [27].

Each item on the Loneliness scale presents three response options, i.e., no, more or less, and yes. For each item, a score of 0 or 1 is given based on the instruction in the instrument. The final score is the total of the score for the 11 items, with the lowest possible score being 0 (which means no loneliness) and the highest being 11 (which means severe loneliness) [28]. Three categories have been proposed based on the score [29]: a score of 0–2 points = no loneliness; a score of 3–8 points = moderate loneliness; and a score of 9–11 points = severe loneliness.

### 2.4. Statistical Analysis

Descriptive statistics, including measurements of the central tendency (mean) and standard error of the mean (SEM) and range values were used to describe all quantitative variables. The normal distribution of each variable was estimated with the Kolmogorov–Smirnov test. Correlation analysis was performed using the Spearman correlation coefficient. Linear regression analysis was used to specify the association between changes in loneliness, and those variables were significant in the previous (bivariate) analyses. The non-parametric Mann-Whitney U test was performed to verify possible differences in the participants’ loneliness scores (emotional, social, and total loneliness score) in relation to socio-demographic factors. Statistical significance was set at *p* < 0.05. Based on the responses of the individuals who participated in the study, an analysis of the reliability of the questionnaire was also carried out by calculating Cronbach’s alpha coefficient. A Cronbach’s alpha value of 0.7 was set as the minimum acceptable value, and lower values indicate low internal consistency of an instrument, although some authors consider that in the early stages of an investigation and in exploratory research, a value between 0.5 and 0.6 may be considered suitable. Statistical analysis was performed using the SPSS 26.0 software package (SPSS Inc., Chicago, IL, USA).

### 2.5. Ethical Approval

All subjects gave their informed consent for inclusion before they participated in the study. The study was conducted in accordance with the Declaration of Helsinki, and the protocol was approved by the Ethics Committee of the University of Valencia (Code: UV-INV_ETICA-2013703 Approval date: 10 January 2022).

## 3. Results

### 3.1. Socio-Demographic Characteristics of the Study Subjects

In total, 530 (84.2% females) community-dwelling individuals from various centers in Valencia and Moncada (Spain) were recruited for this study. In total, 31.7% were widows/widowers, with a mean duration of widowhood of 13.5 ± 0.9 years (0–50 years).

The majority of the subjects did not need a stick or chair to move (79.6%); 17.5% lived in apartments in a building without a lift, and the average floor location in the building was 1.2 ± 0.1 (1st–5th floor). Regarding the civil status of the participants in the study, 56.8% were married, 31.7% were widows/ers, 4.2% were single, and 7% were divorced (and two people (0.4% of the study sample) did not give information related to their civil status). We present the other socio-demographic characteristics that were collected from the study subjects in Table 1.

Further, 41.3% of the subjects used messenger applications (WhatsApp, telegram, or similar) on a daily basis, and 35.5% could make video-calls.

### 3.2. Evaluation of Loneliness and Socio-Demographics

**Figure 1:**
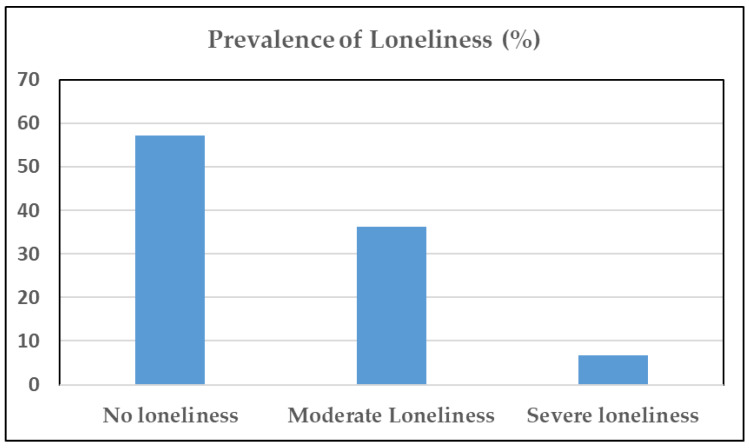
Prevalence of loneliness based on the categorization of the score on the loneliness scale. Score of 0–2 points = no loneliness; score of 3–8 points = moderate loneliness; and score of 9–11 points = severe loneliness.

The average emotional loneliness score was 2.1 ± 0.1 points, the average social loneliness score was 1.3 ± 0.1 points, and the average total score on the loneliness scale was 3.4 ± 0.1 points. Using the cut-off categories of the De Jong-Gierveld Loneliness Scale: from 0 to 2 points means no loneliness, from 3 to 8 points means moderate loneliness, and from 9 to 11 points means severe loneliness; 57.2% of the participants had no loneliness, 36.2% had moderate loneliness, and 6.6% had extreme loneliness (Figure 1). Cronbach’s alpha for the Loneliness scale was 0.82 indicating good reliability for the study samples.

**Figure 2:**
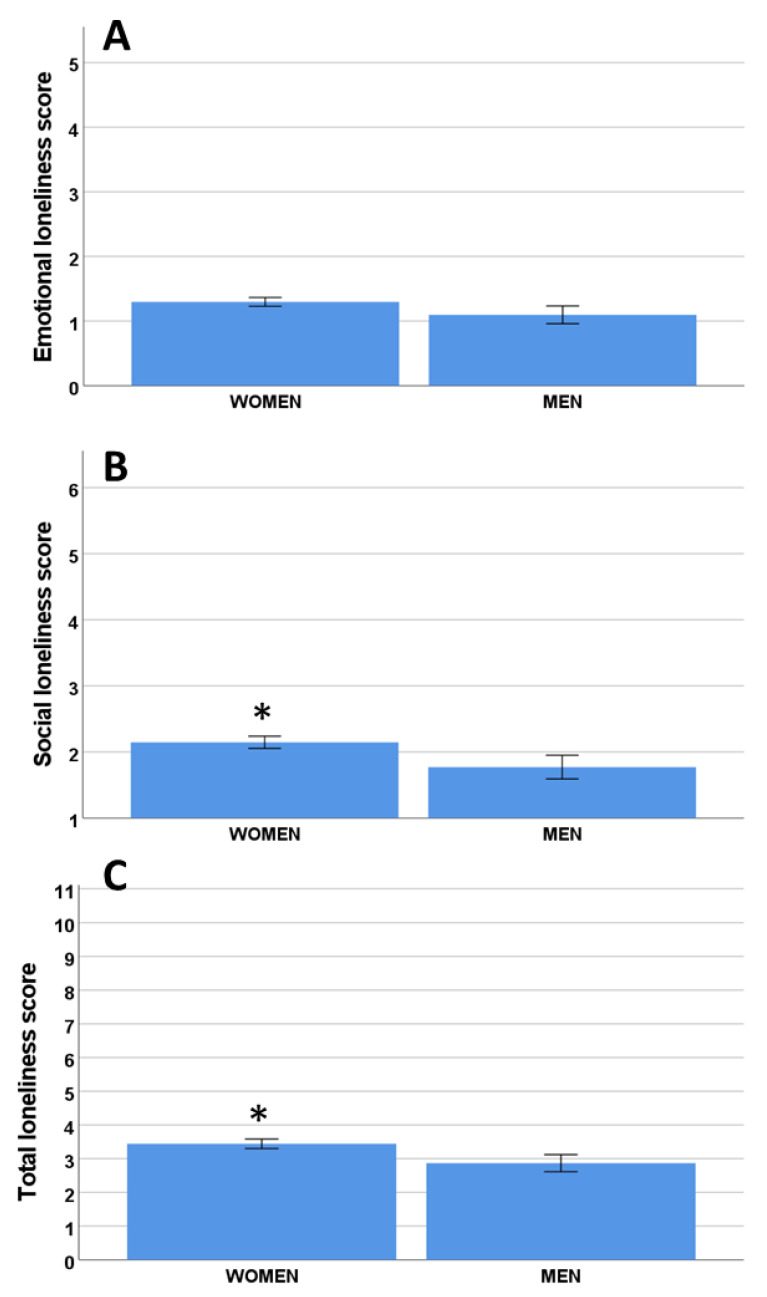
Emotional (**A**), social (**B**), and total (**C**) loneliness scores for men and women. *p* < 0.05. * significant differences.

There were no significant (*p* = 0.09) differences in the emotional loneliness scores between women and men (Figure 2A), but there were significant differences in the social loneliness score (Figure 2B) (*p* = 0.03) and the total loneliness score (Figure 2C) (*p* = 0.03).

**Figure 3:**
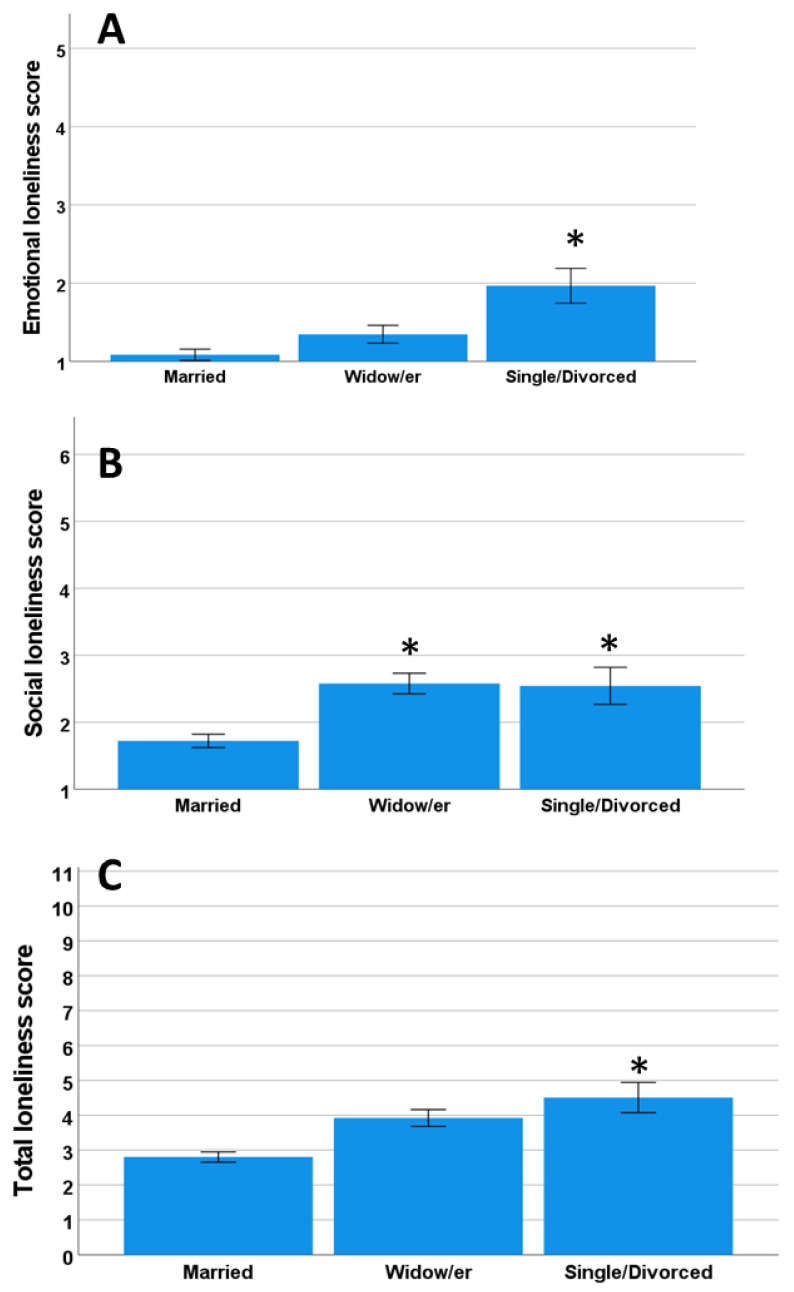
Emotional (**A**), social (**B**), and total (**C**) loneliness scores and marital status. *p* < 0.05 compared to the Married group. * Significant differences.

No significant correlation was observed between age and emotional loneliness (*p* = 0.3), between age and social loneliness (*p* = 0.1), or between age and the total loneliness score (*p* = 0.6). However, there was a significant relationship between marital status and the loneliness score (Figure 3). We categorized marital status into three groups: married, widows/widowers, and single/divorced.

The number of years of widowhood was significantly and inversely correlated with the emotional (*p* = 0.019, rho = −0.201), social (*p* = 0.005, rho = −0.237), and total (*p* = 0.004, rho = −0.248) loneliness scores. Further, 6.2% of individuals who participated in the study provided their contact details, actively asking for help to address their loneliness.

### 3.3. Influence of the Presence of Relatives

**Figure 4:**
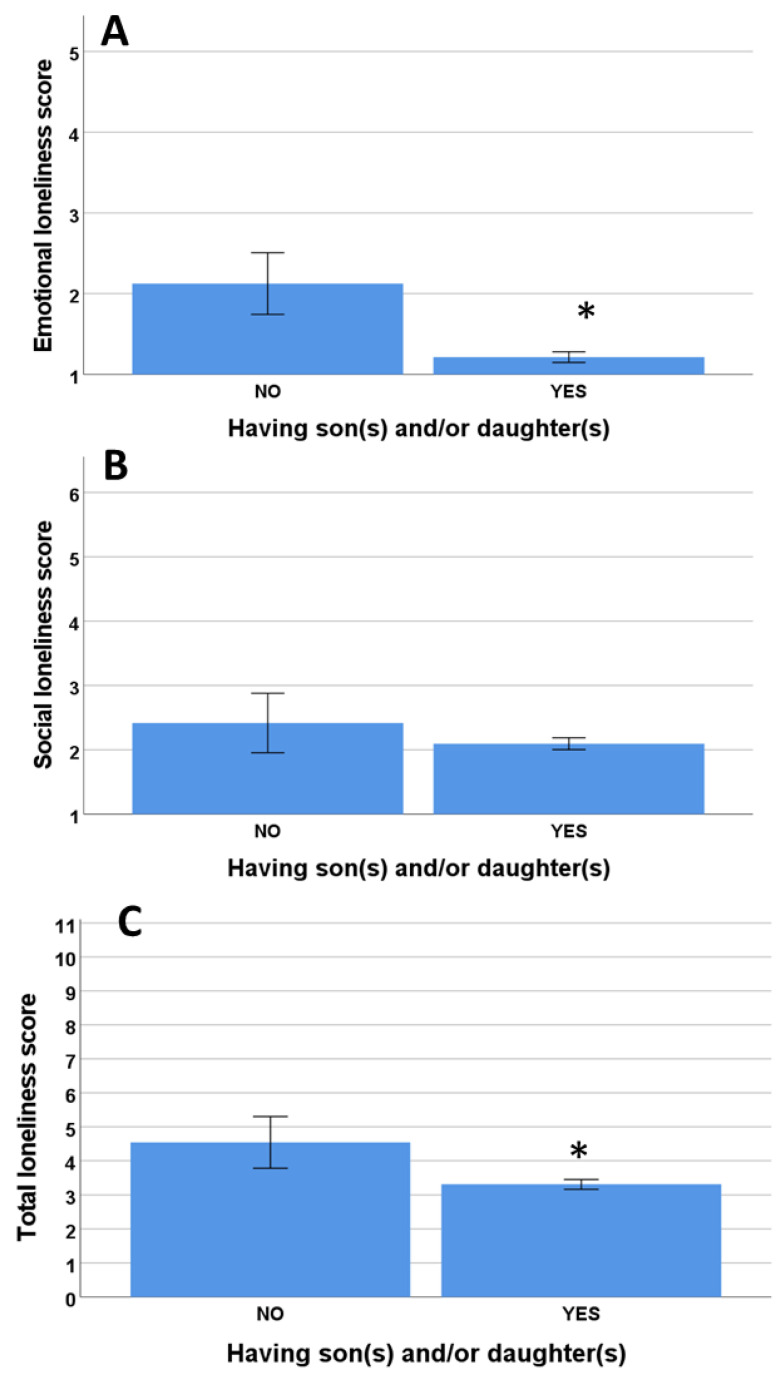
Emotional (**A**), social (**B**), and total (**C**) loneliness scores with and without son(s)/daughter(s). * Significant differences.

The participants with no sons/daughters had significantly higher scores for emotional loneliness compared to the individuals that had sons and/or daughters (Figure 4A) (*p* = 0.007). In contrast, individuals with no sons or daughters had similar levels of social loneliness compared to the individuals with sons/daughters (Figure 4B) (*p* = 0.132). The total loneliness-scale score was significantly higher for individuals with no sons or daughters compared to that for individuals with sons and/or daughters (Figure 4C) (*p* = 0.004).

Individuals with grandchildren did not present with any significant differences compared to individuals with no grandchildren (emotional loneliness *p* = 0.245, social loneliness *p* = 0.884, and total loneliness score *p* = 0.324).

Individuals with siblings did not present with any significant differences compared to individuals without them (emotional loneliness *p* = 0.372, social loneliness *p* = 0.916, and total loneliness score *p* = 0.178).

### 3.4. Loneliness and Impact of Ambulation and Architectonic Barriers

The type of ambulation was not significantly associated with emotional loneliness (*p* = 0.635), social loneliness (*p* = 0.419), or the total loneliness score (*p* = 0.443). Living in an apartment with an elevator in the building had no significant effect on emotional loneliness (*p* = 0.456), social loneliness (*p* = 0.566), or the total loneliness score (*p* = 0.946). Having a mobile phone with an instant messaging service was not statistically significantly associated with any of the types of loneliness, including emotional loneliness (*p* = 0.135), social loneliness (*p* = 0.318), or the total loneliness score (*p* = 0.438). The ability to make video calls with their mobile phone was not statistically significant for emotional loneliness (*p* = 0.649), social loneliness (*p* = 0.293), or the total loneliness score (*p* = 0.300).

A logistic regression analysis was used to determine associations with the variables identified in the bivariate analyses. Selecting the dichotomous variable of the presence of loneliness (loneliness score ≥ 3) or otherwise (loneliness score 0–2) as the dependent variable, we found a significant protective effect of having son(s)/daughter(s) among the participants (*p* = 0.039), with an OR = 0.911 (95% CI 0.809–0.9841), and an opposite effect for those who were divorced/single (*p* = 0.045), with an OR = 1.232 (95% CI 1.04–1.56). Other variables did not show a significant effect.

## 4. Discussion

This study has presented the prevalence of social and emotional loneliness in a Spanish population of older community dwelling-individuals, as well as the impact of sociodemographic variables on the perception of loneliness. The results show a high prevalence of loneliness in this population, with 43% experiencing loneliness to either a moderate (36.2%) or extreme extent (6.6%), compared to similar studies conducted in Spain. For instance, Gené-Badía et al. [30] identified a prevalence of 35% loneliness in an older adult population and a risk of social isolation of 39%, while a study by Olaya et al. [31] concluded that 14.5% of this population felt unwanted loneliness. In this study, 6.2% of individuals provided their contact details, actively asking for help to address their loneliness.

Regarding the high prevalence of loneliness found in this study, it is important to note that the subjects exclusively comprised individuals attending educational and leisure activities organized by senior citizens’ centers. This implies that they were involved in activities that could reduce their feelings of loneliness, as previous research has stated [23]. In future studies, it would be interesting to explore whether attending senior citizens’ centers is a protective factor for loneliness, and if so, it could be recommended by health and social professionals as a strategy to prevent or overcome loneliness in this population.

Unwanted loneliness has been shown to harm individuals’ physical and emotional health [8,10,13,21,32,33]. Indeed, loneliness seems to worsen mental health, increase suicidal ideation, and contribute to unhealthy lifestyles (smoking and alcohol use among others) [4,34]. In specific terms, a Spanish study conducted recently [33] found that individuals who suffered from loneliness have a lower perception of their health status. Other similar studies concluded that there is a relationship between high levels of social loneliness and the risk of depression and frailty [35,36,37,38,39]. These are interesting findings that have not been explored in this study and should be addressed in future research, regarding loneliness as a relevant health variable in itself.

When considering the relationships between sociodemographic characteristics and loneliness, this research focused on age, sex, marital status, family networks, physical barriers and ambulation capacity, and the use of information and communication technology (ICT).

No impact was found with either the total mean age or when stratifying the subjects into two groups according to age (less or more than 70 years of age). This finding contradicts other studies indicating that loneliness and age are associated. For instance, Gené-Badía et al. [30] found that a moderate degree of loneliness is related to aging and suggested that although it could appear at any stage in the lifespan, it is more frequent at advanced ages. The potential association between loneliness and the social network, which is partially lost due to retirement, widowhood, or children becoming independent, could be an explanation for this [22]. However, a cohort study to identify the incidence and determining factors of loneliness in the adult population concluded that the perception of loneliness declines with age [34]. The relationship between age and loneliness therefore seems to be more complex than expected. A meta-analysis conducted on older adults in 2010 identified a U-shaped association between loneliness and age [40]. These controversial results suggest that the relationship between age and loneliness needs to be explored in more detail.

This study identified significant differences in the impact of gender on loneliness. While no differences were found for emotional loneliness, women experienced more social and total loneliness than men. This result supports previous research, which presented a higher prevalence of loneliness in women [21,23,34,41,42]. It is important to stress that the subjects of this study were not representative in terms of gender, as nearly 85% of the participants were women. This is because the participants in the activities organized by the centers are mainly women and is a result of the higher life expectancy of this group. In future research, it would be interesting to further explore the impact of gender on loneliness and the specific reasons why women present with a higher prevalence, especially for social loneliness.

Marital status has widely been considered a factor influencing loneliness. Being married or living with a partner is a protective factor against feeling lonely [2,34,41,43,44]. For instance, Ausín et al. [2] found a higher prevalence of loneliness among single, divorced, and widowed women. A study conducted in China found that married older people reported the lowest levels of loneliness [44]. They explained that the support and company provided by the partner are extremely important in this context due to the fragmentation of the family as a result of the migration of young people and the “one child” policy. The results of the current study support these results, as the married people presented with less loneliness than single, divorced, and widowed people. In specific terms, single people had higher levels of loneliness than their widowed counterparts, and the feeling of loneliness increased when the loss of the partner was recent. Moreover, people with children reported less loneliness, and emotional loneliness in particular, while no differences were identified for social loneliness. Having children was therefore a protective factor against emotional loneliness, and it seems to have no impact on the individuals’ social life or network. This could explain the finding that single people felt more loneliness than widows/widowers, which could be related to the higher probability of the widowed individuals having children than that in the single population. These differences should be explored in detail in future studies.

Meanwhile, having grandchildren, as well as brothers or sisters, did not influence this population’s loneliness. No strong evidence that supports or contradicts this result has been provided to date. A more distant family probably leads to a less cohesive relationship with those relatives, especially in the older adult population, and caring for grandchildren does not impact their feelings of loneliness. Previous research has highlighted the fact that strong family cohesion and frequent weekly contact with family and friends are associated with lower levels of social loneliness [43]. However, aspects, such as whether those relatives live in the same city or the frequency with which they visit, were not measured in this study. All these issues, as well as the differences in the degree of kinship, should be considered in future research to explain these results in more depth.

Another relevant finding of this study is that no relationships were identified between loneliness and the ability to walk or with architectonic barriers at home. These results are inconsistent with previous research, which found a higher prevalence of moderate loneliness among individuals with difficulties in walking and architectonic barriers at home, such as the lack of an elevator [44,45,46,47]. The fact that nearly 80% of the sample was composed of individuals who were completely autonomous in ambulation and who had an elevator probably failed to facilitate a detailed study of these potential differences. 

Results showed that the use of ICT, smartphones, and video calls did not have a significant impact on loneliness. To date, few studies have looked at the use of ICT and loneliness in older adults [24,25,48]. For instance, a cross-sectional study conducted in Germany with this population showed that individuals who used web-connected ICT present with lower levels of loneliness [25]. Furthermore, most intervention studies to increase the use of ICT in older adults reported positive results for loneliness. However, more research, especially randomized controlled trials, is needed on this topic to provide stronger evidence about whether the promotion of ICT in this population could be a useful strategy for reducing loneliness.

This study has some limitations. First, although the sample size is important, a non-probabilistic sampling strategy was used. Moreover, all participants were involved in leisure or educational activities, which could influence their feeling of loneliness. In addition, the fact that the subjects were mostly women, autonomous in their ambulation and without architectonic barriers, made it impossible to explore the impact of those aspects on loneliness in detail. The results therefore cannot be generalized to the older adult population.

It would also have been interesting to explore other sociodemographic variables, such as the level of education, income, and hobbies, in order to be able to better define the profile of older adults that are more vulnerable to loneliness.

## 5. Conclusions

In conclusion, our study complements an extensive body of knowledge about the prevalence of loneliness in older adults and its relationship with some sociodemographic variables. It identified single, separated, or widowed women without children as being the most vulnerable population to loneliness. Unwanted loneliness should be considered a public health issue due to its negative implications for the health of older people. A clear identification of protective and risk factors for loneliness in this population is the first step towards identifying vulnerable individuals and designing and implementing tailored programs to prevent and reduce it.

## Figures and Tables

**Table 1:** Socio-demographic characteristics.

**Sample Size**	530 participants
**Mean Age (Range)**	72.7 ± 0.3 (56–102 years)
**Nationality**	97% Spaniards
**Educational Level**	66% primary education, 44% higher education
**Number of Individuals Living with the Participant (Range)**	1.5 ± 0.04 (0–8 people)
**Sons/Daughters (Percentage/Range)**	81.3% had son(s) and/or daughter(s) Mean 2.2 ± 0.6 (range 0–5)
**Grandchildren (Percentage/Range)**	71.5% had grandchildren Mean 2.8 ± 0.1 (0–12 grandchildren)
**Siblings (Percentage/Range)**	68.1% had siblings Mean 2.2 ± 0.1 (0–9 siblings)

## Data Availability

The data presented in this study are available on request from the corresponding author.
